# Phase separation of the plasma membrane in human red blood cells as a potential tool for diagnosis and progression monitoring of type 1 diabetes mellitus

**DOI:** 10.1371/journal.pone.0184109

**Published:** 2017-09-07

**Authors:** Giuseppe Maulucci, Ermanno Cordelli, Alessandro Rizzi, Francesca De Leva, Massimiliano Papi, Gabriele Ciasca, Daniela Samengo, Giovambattista Pani, Dario Pitocco, Paolo Soda, Giovanni Ghirlanda, Giulio Iannello, Marco De Spirito

**Affiliations:** 1 Istituto di Fisica, Università Cattolica del Sacro Cuore, Rome, Italy; 2 Unità di Sistemi di elaborazione e bioinformatica, Facoltà dipartimentale di Ingegneria, Università Campus Bio-Medico, Rome, Italy; 3 Istituto di Medicina Interna, Fondazione Policlinico Gemelli, Rome, Italy; 4 Istituto di Patologia, Università Cattolica del Sacro Cuore Rome, Italy; Baylor College of Medicine, UNITED STATES

## Abstract

Glycosylation, oxidation and other post-translational modifications of membrane and transmembrane proteins can alter lipid density, packing and interactions, and are considered an important factor that affects fluidity variation in membranes. Red blood cells (RBC) membrane physical state, showing pronounced alterations in Type 1 diabetes mellitus (T1DM), could be the ideal candidate for monitoring the disease progression and the effects of therapies. On these grounds, the measurement of RBC membrane fluidity alterations can furnish a more sensitive index in T1DM diagnosis and disease progression than Glycosylated hemoglobin (HbA1c), which reflects only the information related to glycosylation processes. Here, through a functional two-photon microscopy approach we retrieved fluidity maps at submicrometric scale in RBC of T1DM patients with and without complications, detecting an altered membrane equilibrium. We found that a phase separation between fluid and rigid domains occurs, triggered by systemic effects on membranes fluidity of glycation and oxidation. The phase separation patterns are different among healthy, T1DM and T1DM with complications patients. Blood cholesterol and LDL content are positively correlated with the extent of the phase separation patterns. To quantify this extent a machine learning approach is employed to develop a Decision-Support-System (DSS) able to recognize different fluidity patterns in RBC. Preliminary analysis shows significant differences(p<0.001) among healthy, T1DM and T1DM with complications patients. The development of an assay based on Phase separation of the plasma membrane of the Red Blood cells is a potential tool for diagnosis and progression monitoring of type 1 diabetes mellitus, and could allow customization and the selection of medical treatments in T1DM in clinical settings, and enable the early detection of complications.

## Introduction

Glycosylated hemoglobin (HbA1c) is a marker for average blood glucose levels over the previous three months before the measurement. It is formed in a non-enzymatic glycation pathway by hemoglobin's exposure to plasma glucose. The fraction of glycated hemoglobin increases with the average amount of plasma glucose. The measurement of long-term weighted mean HbA1c is therefore a hallmark of T1DM, and can be employed in diagnostics, therapy monitoring and complications prevention. Nonetheless, in diagnostic settings the measurement of HbA1c does not substitute for standard glucose tolerance testing and, in the absence of overt hyperglycemia, does not adequately discriminate among nondiabetic, diabetic and diabetic with complications [1]. In therapy monitoring, the approach adopted is to keep HbA1c level under a certain threshold, but it is not clear which is the HbA1c level to strive for in the treatment of T1DM [2]. As concerns the complications, long-term weighted mean HbA1c was closely associated with the development of severe complications as cardiovascular disease, nephropathy, neuropathy, and retinopathy [3]. Assessing glycemic control throughout HbA1c level monitoring may prevent complications: the absolute risk of developing them was found to decline with proportional reductions in HbA1c levels [3]. Nonetheless, it is still controversial why patients with low or normal HbA1c levels still develop complications[4,5]. Therefore, the principal concern about HbA1c assay is its low sensitivity in diagnostics, monitoring and complications prevention, as well as its reproducibility, since the wide availability of different methods for HbA1c determination causes over- or underestimation of the amount of HbA1c[6].

To overcome these limits and increase the accuracy of the method, one possible way is to monitor induced modifications on the membrane physical state that alter RBC functions. Glycosylation-induced conformational changes of plasma membrane (PM) and cytosol proteins underline changes in the fluidity of lipid bilayer in diabetes [7–9]. These changes impair several processes, as the glucose transport regulation by insulin, and contribute to the development of T1DM complications[10]. Whether HbA1c level reflects blood glucose levels integrated over three months, RBC membrane fluidity reflects the state of a complex network of regulatory process influenced by the systemic state and the selected therapies, integrated in the same time window: the measurement of these alterations can therefore furnish a more sensitive index of disease progression with respect to the HbA1c level, enabling accurate diagnosis, early detection of complications and the possibility to prevent their development. PM fluidity alterations can be detected by functional two-photon microscopy (fTPM), a method to monitor fluidity[11–14], which can retrieve RBC fluidity maps at submicrometric scale: the fluorescent probe Laurdan, evenly distributed in PM, emits fluorescence whose color depends on the lipid packing of the coexisting areas in PM, allowing a quantitative type of contrast. With this method, solid-ordered and liquid-disordered domains can be distinctly visualized on PM [9,12]. Throughout fTPM the actual length scale of the lipid domains is detectable, allowing the visualization of fluidity patterns dependent on the lipid and protein composition, modulated by the glucose level. Computational methodologies as machine learning (ML) can infer correlations between these patterns and disease progression, in contrast with manual inspection of data that can determine mean values of fluidity, but is unable to unveil hidden information in the RBC fluidity spatial distributions and pattern organization. ML has proven to be effective in catching data not suited for human inspection and in enhancing the discriminative power among cell states. Indeed, it allows making data-driven predictions/decisions on data as it builds a model from example inputs[15], and it has been applied to microscopy images [16–18] and diabetes research[19]. One of the most useful applications of ML in biomedicine consists in the development of decision support systems (DSSs), that are computer-based information systems supporting the decision-making process of the specialists as they can process huge amount of data, being therefore able to overcome the limitations of the direct human data inspection. DSSs have also proven to be effective in a dynamic environment, providing decision in noisy contexts and adapting to various typologies of scenario.

In this work, we report a pilot study for the development of an intelligent platform enabling T1DM diagnosis and progression monitoring. This method, based on the assessment of altered RBC plasma membrane equilibrium, improves the sensitivity of the detection with respect to the traditional HbA1c assay. A DSS to aid the physicians to recognize the presence and the stage of T1DM in a patient given the acquired RBC images is developed and integrated to exploit the full content of the images and to increase the discriminative power among classes.

## Material and methods

All research involving human participants have been approved by the ethical committee of Università Cattolica del Sacro Cuore, Rome, Italy, and all clinical investigation have been conducted according to the principles expressed in the Declaration of Helsinki.

### Selection and classification of patients

For this case-control study 26 subjects, 18 with T1DM and 8 healthy control (group G0), were enrolled. Subjects with T1DM were classified in subjects without complications and short duration of disease (<15 years) (n = 11, group G1) and subjects with complications and long duration of disease (≥15 years) (n = 7, group G2). Subjects in the three groups were matched for age and BMI. Subjects with T1DM were matched also for metabolic control, defined by glycosylated hemoglobin (HbA1c) levels. For G1 and G2 groups inclusion criteria were diagnosis of Type 1 DM, age ≥ 18 years old. For all groups exclusion criteria were: diagnosis of type 2 diabetes, previous pancreatic surgery or chronic pancreatitis, medical history of cancer in last five years prior the enrollment; presence of any blood dyscrasia causing hemolysis or unstable red blood cells.

Statistical T-tests for sets of biological/biophysical data are performed by Microsoft Excel. Values of p< 0.05 are considered significant. Other kinds of analysis are performed as outlined in the machine learning section.

### Blood cells extraction and selection

RBC are extracted as in Hanson et al.[20], seeded on a multi-well plate and directly visualized on the microscope in a sterile 0.9% NaCl solution.

### Membrane fluidity measurements on red blood cells

Cells were imaged with TPM (under excitation at 800 nm with a mode-locked Titanium-Sapphire laser, Chamaleon, Coherent, Santa Clara, CA) within one hour from extraction. 1 μl of Laurdan 1 mM stock solution (Molecular Probes, Inc., Eugene, OR, USA) were added per milliliter of Dulbecco's Modified Eagle's Medium (DMEM). Laurdan intensity images were recorded simultaneously with emission in the range of 400–460 nm and 470–530 nm and imaging was performed at 37°. All images were acquired at 200 nm pixel resolution (60X objective). Background values were measured and subtracted for each image, and debris or other aggregates were removed to avoid biases in the analysis. As a normalized ratio of the intensity at the two emission wavelengths regions, the generalized polarization (GP) provides a measure of membrane order, in the range between -1 (liquid-crystalline) and +1 (gel). The GP index

$$GP=\frac{I_{\left(400-460\right)}-GI_{\left(470-530\right)}}{I_{\left(400-460\right)}+GI_{\left(470-530\right)}}$$ (1)

was calculated for each pixel using the two Laurdan intensity images (I (400–460) and I (470–530)) by using the program Ratiometric Image processor[21]. This index is independent from excitation intensities, probe concentrations and other artifacts since laurdan it is a ratiometric probe.

### Machine learning

We investigate how a DSS can be employed to assign an unknown patient to one of the three aforementioned groups (named as G_0_, G_1_, and G_2_). Formally, suppose we are given a set of training data (**x**_1_, c_1_), …, (**x**_n_, c_n_), where the input (also known as feature vector) **x**_i_ ∈ R^p^, and the output (response variable) c_i_ is qualitative and assumes values in the finite set {G_0_, G_1_, G_2_}. A DSS consists either in single classification rule C(**x**) or in a set of classification rules Cj(**x**), with j = 1,…, m, optimized on a set of training data to maximize/minimize an objective function so that when given a new input **x**, the DSS can assign it a class label c from {G_0_, G_1_, G_2_}.

In other words, a classifier C(**x**) builds a decision boundary partitioning the feature space R^p^ in decision regions, one per class. The form of such boundary depends upon the classification rule employed.

The DSS we developed firstly computes a set of features from each sample image acquired in the red and the green channels at different z-axis position. A set of features is computed from the whole image. The features consist of heterogeneous descriptors integrating information of different nature as they compute texture measures based on statistical descriptors and rotation invariant co-occurrence local binary patterns. The former consists in first and second-order measures (e.g., moments, gray level co-occurrence matrix)[22], whereas the latter is a descriptor assigning to each pixel of the image a label obtained comparing it with its neighborhood matrix[23]. The DSS also leverages on the Principal Component Analysis (PCA), which is a statistical procedure that uses an orthogonal transformation to convert a set of features of possibly correlated variables into a set of linearly uncorrelated variables called principal components. The number of principal components is less than or equal to the number of original features. This transformation is defined in such a way that the first principal component has the largest possible variance (that is, accounts for as much of the variability in the data as possible), and each succeeding component in turn has the highest variance possible under the constraint that it is orthogonal to the preceding components[24]. The classification stage of the DSS assigns each image of a patient a label c_i_ using a Support Vector Machines[25]. Next, the labels assigned to the images of the stack are combined to assign a label to the patient. The cardinality of the patient dataset suggested us to conduct all the experiments using the leave-one-person-out (LOPO) approach, i.e. a variant of cross-validation method which keeps one patient in the test set and it uses the remaining observations as the training set. In other words, for each iteration of the LOPO approach, data of a single patient are used to validate the algorithm, whereas data of all the other patients are used to train the algorithm. According to this procedure, for each iteration, the algorithm is tested on a set separated from the training one, avoiding any bias. Finally, the performance for all the folds of the LOPO approach are averaged out. Furthermore, we also would like to highlight that the PCA was also run for each training iteration of the LOPO approach: indeed, once the new features were computed, test images are projected into the PCA space and the selected components are retained. Again, this procedure avoids any bias when estimating the performance. The decisions of the two pipelines are aggregated in the combiner block exploiting the multi-spectral information which takes the final decision using the weighted voting (WV) rule combining the classification outputs provided by two classification systems.

We also developed a Bayesian classifier leveraging on HbA1c values as we are interested in comparing the recognition performance of the proposed DSS with those achieved by an approach based on HbA1c. To this end, the Bayesian approach divides the subjects into three groups using the statistical distributions of the HbA1C level. Again, the experiments were run adopting the leave-one-person-out approach.

Furthermore, we also compared our DSS with an approach that classifies the subjects using only the average scalar score computed from the GP image $\left(\bar{GP}\right)$ that, hence, it does not employ multispectral information. Again, we adopted a Bayesian approaches and the leave-one-person-out approach.

## Results

### Study population

S1 Table describes the characteristics of the 26 participants. All subjects were comparable for age, body mass index (BMI), waist and hip circumference, lipid profile and creatinine. Subjects with diabetes were comparable for glycated hemoglobin and insulin requirement, defined as total daily insulin dose/weight. Subjects with diabetes were significantly different for duration of disease (p<0.001).

### RBC fluidity is not homogeneous, and it is organized in macro-domains

**Fig 1:**
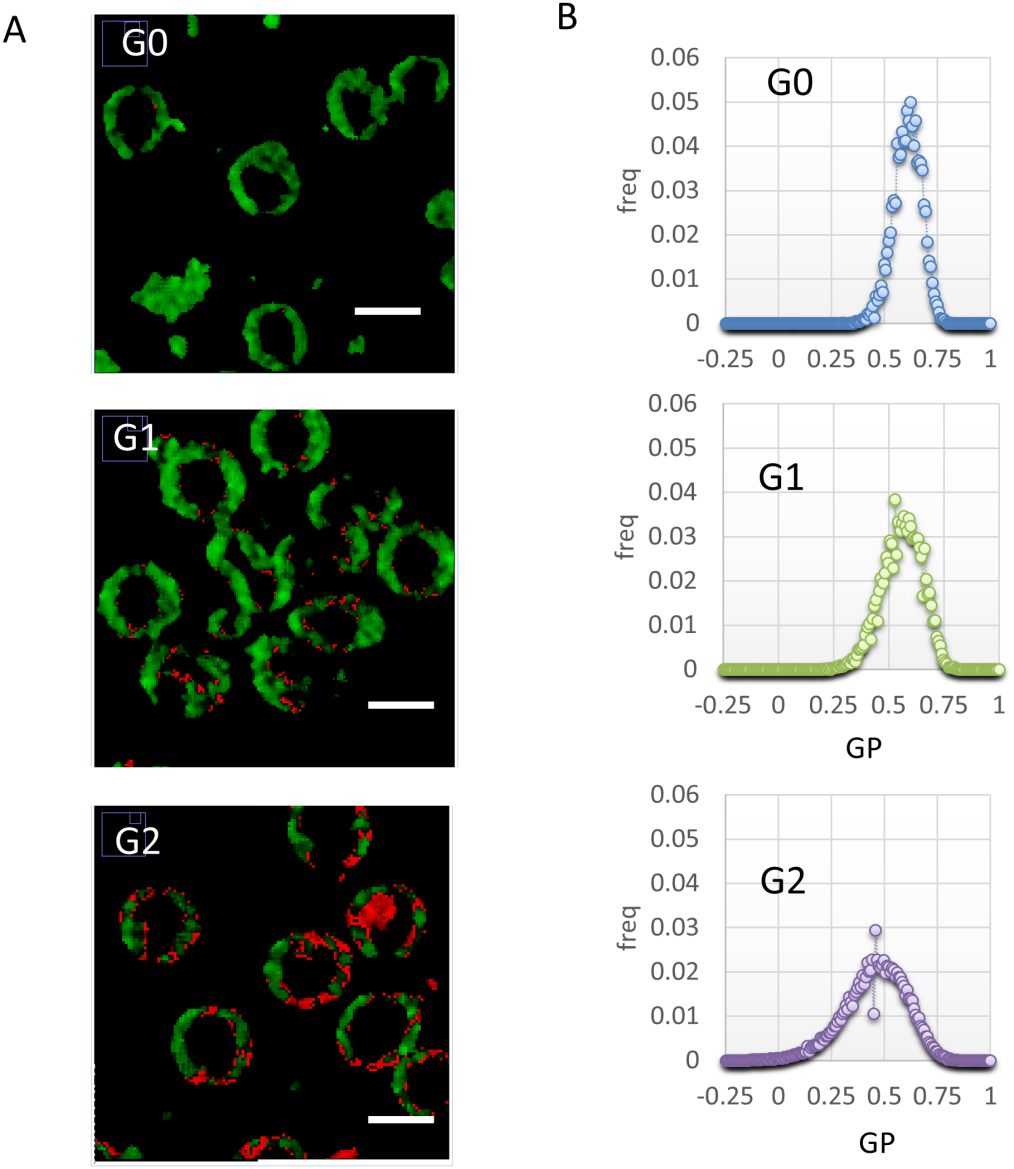
Sub-micrometric spatial distribution of RBC fluidity. (A) Submicrometric fluidity maps of RBC are reported, in a two-colored scale spanning from red (liquid-like) to green(gel-like). G0: control; G1: T1DM, no complications; G2: T1DM with complications. (B) GP Histograms of the corresponding images, showing a shift of the mean value towards more fluid regions going on with disease progression, but also an increase in variance.

Representative submicrometric fluidity maps of RBC are reported in Fig 1A, in a two-colored scale spanning from red (liquid-like) to green (gel-like). An inhomogeneous domain organization is evident in the different groups monitored (G0: control; G1: T1DM, no complications; G2: T1DM with complications). Since RBC have not interior organelles, Laurdan labels just the plasma membrane, and the fluidity outcome corresponds directly to PM fluidity. Fluid clusters begin to appear on PM in the G1 patients, suggesting that a phase separation is occurring. The mean dimension of these clusters is 500 nm-1μm. In the G2 patients, fluid domains increase in number and dimensions, and there is still a phase separation. The different membrane organization of the three groups is evident from the shift of the GP distributions mean value and the increase in variance as the disease progresses (Fig 1B).

### Comparison between GP and HbA1c level sensitivity

**Fig 2:**
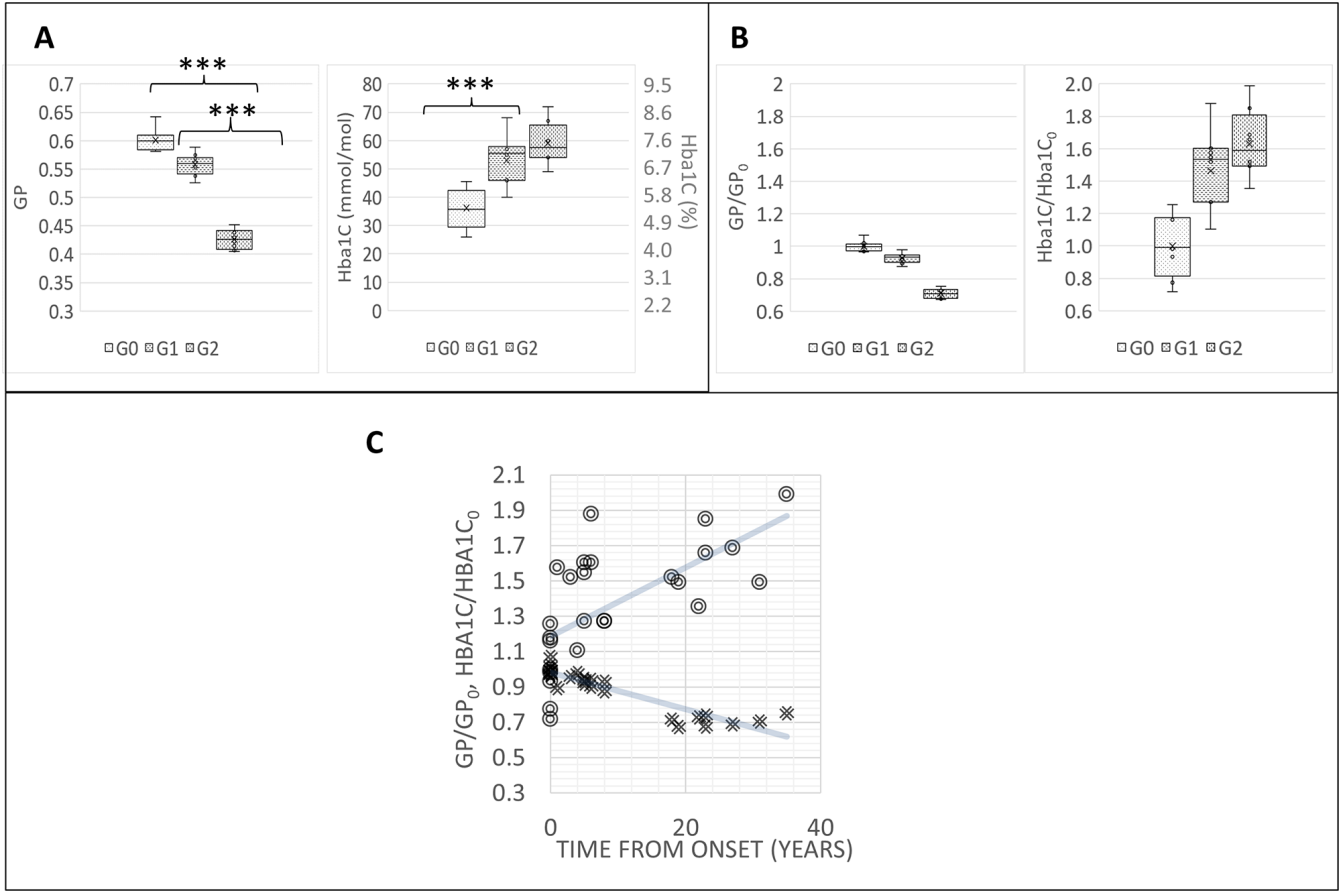
RBC fluidity allows to classify patients with and without complications, and increases with time after the first diagnosis. (A) Mean GP values and HbA1c values of different groups G0: control; G1: T1DM, no complications; G2: T1DM with complications. *** stays for p<0.001. (B) Mean GP values and HbA1c values normalized to GP_0_ and HbA1c_0_ values (average over the G0) of different subgroups. (C) Mean GP values and HbA1c normalized values of RBC fluidity variations with time after the first diagnosis. Solid lines represent linear regression curves. The slopes of the Hba1C/Hba1C_0_ curve is 0.0195 y^-1^, while the slope of the GP/GP_0_ is -0.0104 y^-1^. R^2^ values are respectively 0.4213 and 0.8365 for the Hba1C/Hba1C_0_ and GP/GP_0_.

In Fig 2A, GP values are reported for each analyzed group. GP values show significant differences (p<0.001) among the groups. HbA1c values present significant differences only between G0 and G1 patients. In Fig 2B, the same values normalized to GP_0_ and HbA1c_0_ values (averages over the G0) show graphically that the coefficient of variation of the GP data, which represents the extent of variability in relation to the mean of the population, is about 0.03, whether the coefficient of variation of HbA1c_0_ is much higher, about 0.2. In Fig 2C, the same normalized values of RBC fluidity increase (i.e.GP decreases) with time after the first diagnosis. The decrease is monotonic for 10 years, then a plateau is reached at 20 years. The increase in HbA1c is again characterized by a higher variability than GP: by performing a linear regression of the curves, R^2^ values are respectively 0.4213 and 0.8365 for the Hba1C/Hba1C_0_ and GP/GP_0_, further confirming that GP measurements show less variability than the HbA1C measurements.

### DSS augments discriminative power among the three groups

A machine learning approach was employed to aid the physician to recognize the group of a patient, given the acquired RBC images. This system, exploiting the full content of the images, increase the discrimination power among classes. As reported in the methods, the PCA converts the set of features computed from the images, which should be correlated into a set of linearly uncorrelated variables called principal components. Furthermore, it is well-known that a limited, yet salient, feature set simplifies both the pattern representation and the classifiers that are built on the selected representation, as it alleviates the curse of dimensionality. For this reason, the whole set of principal components was reduced to 31 descriptors, which map the 99% of the variance. We compared three different DSS: a Bayesian approach based either on the HbA1c values or on the average values of GP and a DSS which integrates information computed from the two channels of the color images, thus leveraging multi-spectral data embedded in the images. The latter obtains large and stable recognition performance (Table 1). As reported in the last row of Table 1, the DSS provides the best classification into three classes of the subjects involved in the study, suggesting that it can augment the discriminative power among the groups. The performance was evaluated throughout *Accuracy*, *Precision*, *Recall* and *F1* parameters, which are commonly used to quantify the global ability to correctly perform the classification task(Table 1 and S2 Table). The second and third rows of the Table 1 show the performance achieved by the Bayesian approach based either on the HbA1c values or on the average values of GP.

**Table 1:** Performances of the tested methods in classes recognition task: G0(Control), G1 (T1DM) and G2 (T1DM with complications).

		G0			G1			G2		
	*Accuracy*	*Precision*	*Recall*	*F1*	*Precision*	*Recall*	*F1*	*Precision*	*Recall*	*F1*
*HbA1c*	0.54	0.88	0.88	0.88	0.40	0.40	0.40	0.38	0.38	0.38
*GP*	0.88	0.75	0.86	0.80	0.90	0.82	0.86	1.00	1.00	1.00
*DSS*	1.00	1.00	1.00	1.00	1.00	1.00	1.00	1.00	1.00	1.00

Referring to S2 Table, the parameters among the columns are: *Precision*–the fraction of the correctly classified subjects respect to the number of subjects classified as belonging to that class, (A/(A + B + C) for class 1); *Recall*–the fraction of the correctly classified subjects respect to the number of subjects effectively belonging to that class (A/(A + D + G) for class 1); *f1 msr*–the harmonic mean between Precision and Recall (2*(Precision*Recall)/(Precision + Recall)); *Accuracy*–a classifier score to quantify its global ability to correctly perform the classification task ((A + E + I)/(∑(A to I))).

### Blood cells cholesterol increases with RBC fluidity

**Fig 3:**
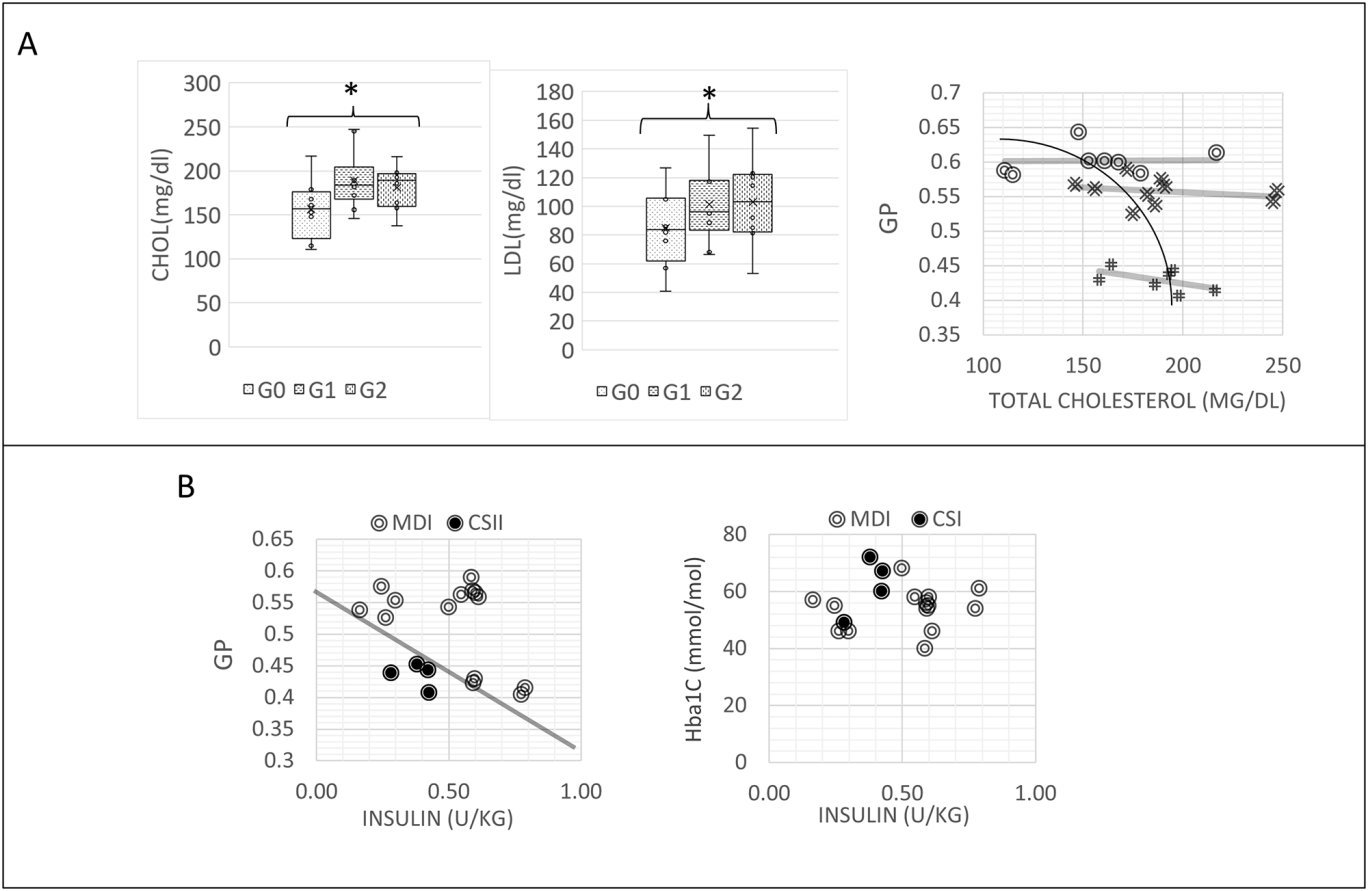
Blood cells cholesterol and LDL increases with RBC fluidity and RBC fluidity increases with administered insulin. (A) Plasma cholesterol content and LDL content in the different groups. G0: control; G1: T1DM, no complications; G2: T1DM with complications, and relationship between plasma cholesterol content and RBC membrane fluidity in the different groups. * stays for p<0.05. The variation inside group G1 and G2 correspond to an increase of fluidity with cholesterol and LDL content, as evident from the negative slopes of the linear regressions to the data, which are for G0, G1 and G2 respectively 0.0002, -0.0001, -0.0004. the low R^2^ values (respectively 0.0829, 0.0587 and 0.2788 for G0, G1 and G2) indicate that, apart cholesterol, the linear model has to account for other factors. (B) RBC fluidity is correlated with the units of insulin administered pro Kg. Relationships are different for patients undergoing different intensive insulin therapies: multiple daily injections(MDI) or insulin pump (CSI). The solid line is traced to show the possibility to separate the two groups of data in the plane.

Free cholesterol in RBC plasma membranes can exchange bidirectionally with plasma lipoprotein cholesterol [26,27], therefore we can assume that plasma cholesterol content reflects RBC cholesterol content. According to our analysis, blood cholesterol content increases, and the difference is significant between group G0 and group G2 (Fig 3A). Total cholesterol content is inversely correlated with RBC fluidity values (Fig 3A). Also the intra-group variation in G1 and G2 shows an increase of fluidity with cholesterol and LDL content, as evident from the negative slopes of the linear regressions to the data, which are for G0, G1 and G2 respectively 0.0002, -0.0001, -0.0004 dl/mg. the low R^2^ values (respectively 0.0829, 0.0587 and 0.2788 for G0, G1 and G2) indicate that, apart cholesterol, the linear model has to account for other factors. (Fig 3A). LDL content increases following the same trend (Fig 3A).

### RBC fluidity allows for a better discrimination of therapies

RBC fluidity is correlated with the units of insulin administered pro Kg (Fig 3B). Relationships are different for patients undergoing different intensive insulin therapies: multiple daily injections (MDI) or insulin pump (CSII). MDI patients display normal values of fluidity, until 0.60 U/kg is reached. For higher values, a drop off of fluidity is observed. CSI patients are characterized by higher values of fluidity (lower GP), also at values lower than 0.60 U /Kg. These different relationships can be exploited in discriminating the different therapies according to their direct and indirect effects on membrane fluidity (line in Fig 3B). By using HbA1c, it is more difficult to separate these contributions(Fig 3B).

## Discussion and conclusions

**Fig 4:**
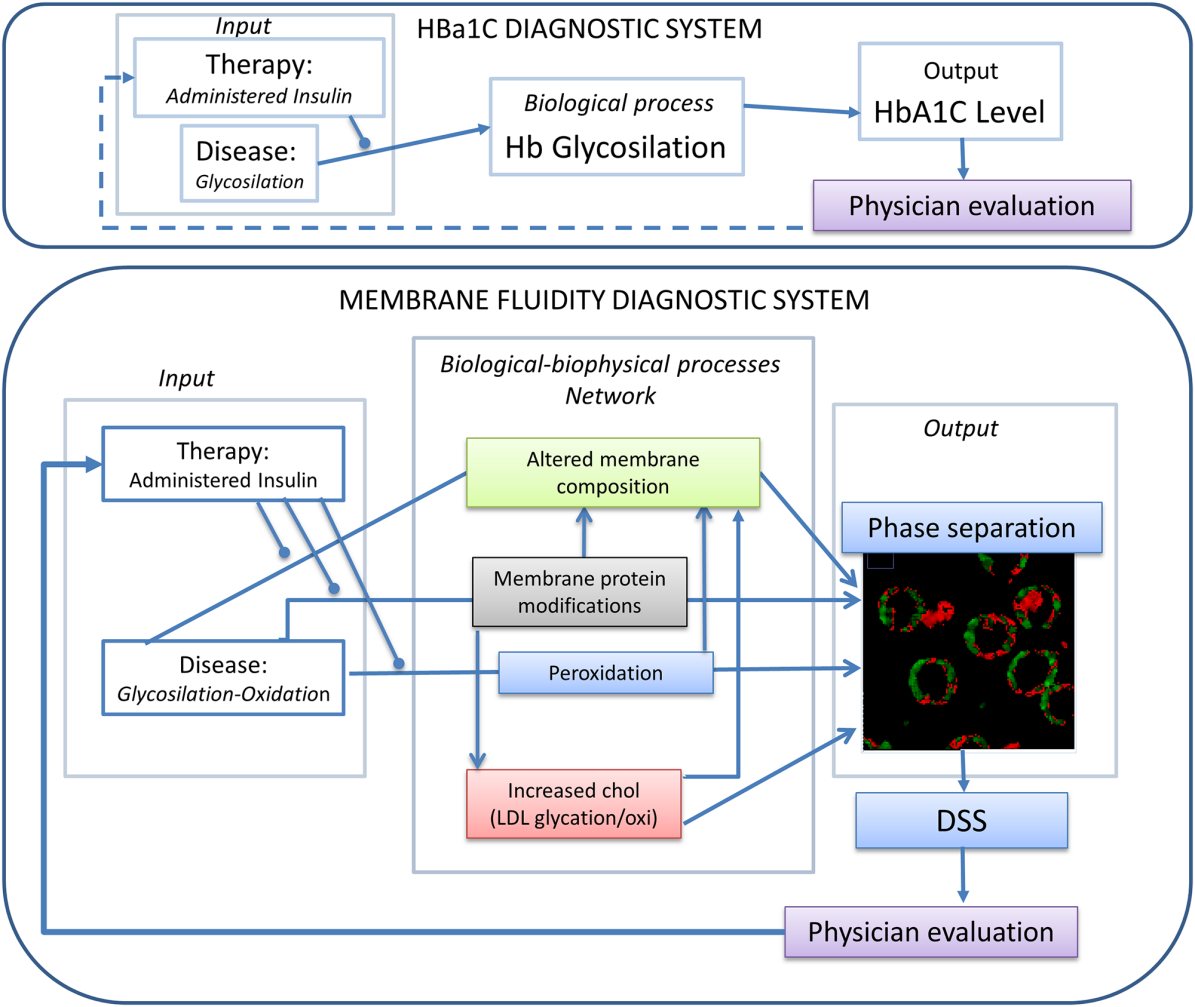
Glycosylation and oxidation, expression of the systemic state, and insulin-dependent membrane remodeling, related to the therapy adopted, can lead to RBC fluidification and phase separation in T1DM.

In this work, a pilot study for a machine-learning based fTPM approach for diagnosis and monitoring of T1DM presented. RBC membrane physical state, showing pronounced alterations in T1DM, is the ideal candidate for monitoring the disease progression. We observed an increasing fluidification of the RBC membrane with the progression of the disease, and a separation and a formation of microdomains with different fluidity. From the sub-micrometric fluidity maps is possible to observe a widening of the fluidity spectrum, with a consistent increase of the fluid region. This biological and biophysical process is exploited to develop a diagnostic system, which is a protocol for measuring a variation of a measurable quantity (*output*) induced in an organism by the disease (*input*). The relation between *input* and *output*, and the sensitivity and the accuracy of the system, depends on the monitored biological and biophysical processes which occurs in the diagnosed organism. In the case of HbA1c assay, the biophysical process of the diagnostic system linking the *input* (T1DM state of the disease) to the *output* (HbA1c level) is the glycosylation of hemoglobin (Fig 4). In the case of the fluidity-based diagnostic system, the *output* is the variation in membrane fluidity, and the biophysical process connecting input and output consists in a network of systemic effects related to the chronic hyperglycemia, oxidative stress and metabolic alterations triggered by absolute insulin deficiency (glyco-oxidation), rather than the glycosylation of a single protein (Fig 4). These conditions are a source of permanent, cumulative damage to RBC membranes in diabetes, integrated over an average period of three months. Indeed, RBC are uniquely vulnerable to these effects, due to their inability to synthesize new proteins and degrade altered, non-functional proteins[28]. Glycosylation and other post-translational modifications of membrane and transmembrane proteins can alter lipid density, packing and interactions, and are considered an important factor that affects fluidity variation in membranes and organelles[28]. It was observed that in T1DM the fluidification was concomitant with a decrease of AchE and Na+,K+-ATPase activities[29]. Also the LDL-receptor-mediated clearance mechanisms could be impaired, and may contribute, as we detected in group G1 and G2 patients, to elevated cholesterol and LDL levels in patients with diabetes, with an impact on RBC plasma membrane lipid composition [14]. Other studies support these findings: the rise of LDL in plasma of diabetic patients [30], and a significant rise in phospholipids and cholesterol, as well as levels of lipid peroxidation products, are detected in RBC membrane of T1DM and T2DM [31–33]. Small-angle X-ray diffraction indicated that high concentration of cholesterol in plasma membranes, self-assemble to form immiscible domains [34]. Increase in cholesterol content means also an increased stiffness. This change in viscoelastic properties of RBC membranes in type 2 diabetes was indeed already monitored by using atomic force microscopy by our group[35]. On the other hand, non-enzymatic peroxidation transforms polyunsaturated fatty acids (PUFA), into oxidized metabolites, altering membrane composition and properties[36,37]. An occurrence of gel-like lipid regions was observed and mainly attributed to augmented lipid peroxidation[7,9]. Lipid peroxidation generates bioactive compounds such as α,β-unsaturated aldehydes (for example, 4-hydroxynonenal), that can react rapidly and non-enzymatically with electronegative moieties in biomolecules and form covalent adducts, alter their function and cause cell damage [9,38–41]. Insulin treatments, by influencing the state of the network (Fig 4) can alter this fluidification effect as observed on T1DM patients. We observed that therapies that vary the dose as well as the way of administration (CSI or MDI) alter the fluidity of RBC membranes in different ways and their effect can be clearly distinguished.

These three outlined mechanisms: glycosylation and oxidation, expression of the systemic state, and therapy-dependent membrane remodeling, can lead, throughout the outlined integrated network, to RBC fluidification and phase separation in T1DM (Fig 4). The observed mean GP variations allows a discrimination among non-diabetic, diabetic, and diabetic with complications patients. This pilot study demonstrates that the measurement of membrane physical state in these cell types is an ideal target to increase sensitivity in monitoring disease progression with respect to traditional assays as HbA1c, by increasing the accuracy of T1DM diagnosis (a classifier score to quantify its global ability to correctly perform the classification task) from 0.54 (HbA1c) to 0.88 (GP) (Table 1). A simple measurement of RBC in a cuvette could be enough to relay therapy assessments upon mean values. However, the spatial resolution provided by the two-photon technique is plenty of additional information: fluid clusters begin to appear throughout the membrane in the G1 patients (Fig 1B), increasing in number and dimensions in G2. To exploit the information embedded in the images and enhance the sensitivity a machine learning approach was used to discriminate patients into three groups. We developed a DSS employing the full multi spectral content embedded in the images via a set of specific features, which provides larger recognition performance than those attained by using the HbA1c values or an average measure of fluidity. The development of this DSS increases the accuracy from 0.54(HbA1c) to 1.00. Indeed, computing a single score from the GP images, although useful for human inspection, removes useful information embedded in the color channels of the raw images.

This pilot study outlined the possibility to monitor T1DM progression with the introduction of innovative methods based on membrane fluidity determination. These will improve the decisional process in terms of prevention and treatment offer, and it will test and demonstrate new models and tools for health and care delivery, i.e. detection of membrane fluidity at nanoscale by TPM. The main limitation of this study is in the number of subject analyzed, and to overcome this point future efforts will be directed towards the validation of the approach on a larger population. Potential bias could arise in the analysis of other environmental factors that could change the fluidity of the membrane in a similar way. However, the possibility to acquire sub-micrometric maps and the analysis via the ML algorithm can potentially distinguish different phase separation patterns induced by different causes. Once validated, this approach has the potential to be used as a clinical assay, since, after blood extraction, commercial image-based high-throughput systems can analyze multiwell plates providing computer-controlled, automatic image capture and analysis. Analyzed Data could be then collected via a unified web service reducing the number of developed complications will lead to a decrease of administrative, handling and healthcare costs.

Indeed, in the transition to precision medicine, the development of this personalized, high throughput, healthcare platform which manages, integrates, and analyzes chemical and biophysical parameters extracted from blood cell membranes may allow customization and the selection of medical treatments in T1DM in clinical settings, as well as to enable the early detection the complications and to forecast their development. Finally, it can have the potential to be integrated in other diagnostic networks, which collect inputs from other clinical and imaging methods and it is applicable to screen other pathologies involved in blood cell membrane deregulation (i.e. metabolic diseases, cancer).

## Supporting information

S1 Table Characteristics of the 26 participants. All subjects were comparable for age, body mass index (BMI), waist and hip circumference, lipid profile and creatinine. Baseline characteristics between groups have been compared with ANOVA for parametric variables and χ2 for non parametric variables. All groups were comparable except for creatinine.
(DOCX)

S2 Table 3 classes confusion matrix. Each letter at row i and column j represents the number of subjects classified as class i and effectively belonging to class j.
(DOCX)
