# Supplementary material for: Phase separation of the plasma membrane in human red blood cells as a potential tool for diagnosis and progression monitoring of type 1 diabetes mellitus
Source: PLoS One. 2017 Sep 7;12(9):e0184109. doi: 10.1371/journal.pone.0184109 (PMC5589169; doi:10.1371/journal.pone.0184109)
Supplement: S1 Table — All subjects were comparable for age, body mass index (BMI), waist and hip circumference, lipid profile and creatinine. Baseline characteristics between groups have been compared with ANOVA for parametric variables and χ2 for non parametric variables. All groups were comparable except for creatinine. (DOCX) [file pone.0184109.s001.docx]

| **Variable** | **G0 (n=8)** | **G1 (n=11)** | **G2 (n=7)** | **P value** |
| --- | --- | --- | --- | --- |
| Age (years) | 34±9 | 38±9 | 40±9 | 0.52 |
| BMI (kg/m^2^) | 23.1±2.4 | 21.9±3.4 | 24.7±2.7 | 0.17 |
| Waist circumference (cm) | 78±7 | 79±10 | 84±11 | 0.41 |
| Hip circumference (cm) | 94±5 | 97±8 | 98±8 | 0.61 |
| Years of Diabetes (years) | n.a. | 6±5 | 25±3 | <0.001 |
| HbA1c (mmol/mol) | 36±7 | 53±8 | 60±8 | 0.94 |
| UI/Kg | n.a. | 0.47±0.06 | 0.50±0.06 | 0.62 |
| Total Cholesterol (mg/dL) | 156±34 | 184±35 | 187±20 | 0.12 |
| HDL Cholesterol (mg/dL) | 58±11 | 72±15 | 62±14 | 0.07 |
| Triglycerides (mg/dL) | 67±32 | 73±54 | 76±25 | 0.91 |
| Creatinine (mg/dL) | 0.85±0.14 | 0.64±0.12 | 0.80±0.15 | 0.001 |

S1 TABLE. Characteristics of the 26 participants. All subjects were comparable for age, body mass index (BMI), waist and hip circumference, lipid profile and creatinine. Baseline characteristics between groups have been compared with ANOVA for parametric variables and χ^2^ for non parametric variables. All groups were comparable except for creatinine.
