# Supplementary material for: Phase separation of the plasma membrane in human red blood cells as a potential tool for diagnosis and progression monitoring of type 1 diabetes mellitus
Source: PLoS One. 2017 Sep 7;12(9):e0184109. doi: 10.1371/journal.pone.0184109 (PMC5589169; doi:10.1371/journal.pone.0184109)
Supplement: S2 Table — Each letter at row i and column j represents the number of subjects classified as class i and effectively belonging to class j. (DOCX) [file pone.0184109.s002.docx]

|  | Effective class 1 | Effective class 2 | Effective class 3 |
| --- | --- | --- | --- |
| Estimated class 1 | A | B | C |
| Estimated class 2 | D | E | F |
| Estimated class 3 | G | H | I |

**S2 Table**. 3 classes Confusion Matrix. Each letter at row i and column j represents the number of subjects classified as class i and effectively belonging to class j.
